# Certainties and Uncertainties of Cardiac Magnetic Resonance Imaging in Athletes

**DOI:** 10.3390/jcdd9100361

**Published:** 2022-10-20

**Authors:** Liliana Szabo, Giulia Brunetti, Alberto Cipriani, Vencel Juhasz, Francesca Graziano, Kristof Hirschberg, Zsofia Dohy, Dorottya Balla, Zsofia Drobni, Martina Perazzolo Marra, Domenico Corrado, Bela Merkely, Alessandro Zorzi, Hajnalka Vago

**Affiliations:** 1 Heart and Vascular Centre, Semmelweis University, 1122 Budapest, Hungary; 2 Department of Cardiac, Thoracic and Vascular Sciences and Public Health, University of Padova, 35122 Padova, Italy; 3 Department of Sports Medicine, Semmelweis University, 1122 Budapest, Hungary

**Keywords:** athlete’s heart, cardiovascular magnetic resonance imaging, physiological adaptation, cardiomyopathies

## Abstract

Prolonged and intensive exercise induces remodeling of all four cardiac chambers, a physiological process which is coined as the “athlete’s heart”. This cardiac adaptation, however, shows overlapping features with non-ischemic cardiomyopathies, such as dilated, arrhythmogenic and hypertrophic cardiomyopathy, also associated with athlete’s sudden cardiac death. Cardiac magnetic resonance (CMR) is a well-suited, highly reproducible imaging modality that can help differentiate athlete’s heart from cardiomyopathy. CMR allows accurate characterization of the morphology and function of cardiac chambers, providing full coverage of the ventricles. Moreover, it permits an in-depth understanding of the myocardial changes through specific techniques such as mapping or late gadolinium enhancement. In this narrative review, we will focus on the certainties and uncertainties of the role of CMR in sports cardiology. The main aspects of physiological adaptation due to regular and intensive sports activity and the application of CMR in highly trained athletes will be summarized.

## 1. Introduction

Cardiac adaptation due to regular and intense exercise is a well-known phenomenon leading to symmetric hypertrophy and volumetric adaptation summarized by the term “athletes’ heart” [1]. The intensive training-induced enlargement of all four cardiac chambers is a well-known attribute of cardiac adaptation. However, it shows overlapping features with important cardiovascular diseases such as dilated (DCM), arrhythmogenic (ACM) and hypertrophic (HCM) cardiomyopathy [2].

Critically, these diseases are among the most important underlying alterations leading to rare (approximately 1 in 50,000) but tragic and highly publicized events of sudden cardiac death (SCD) among young athletes [3,4]. Although up to 80% of athletes are asymptomatic before major adverse cardiovascular events, sophisticated screening tools in a serial fashion can uncover predisposing factors, leading to early diagnosis and target interventions potentially preventing SCD.

Cardiac magnetic resonance (CMR) imaging metrics show high reproducibility and the method is well-suited to differentiate physiological adaptation from pathological alterations [5]. CMR allows accurate characterization of the morphology and function of the cardiac chambers, providing full coverage of the ventricles. Using specific techniques and gadolinium-based contrast material, CMR can also provide tissue level information including myocardial necrosis, fibrosis or edema [6].

To distinguish between physiological and pathological remodeling with high precision, we must establish what normal means within the context of the athlete’s heart. Therefore, we summarize the main aspects of physiological adaptation due to regular and intensive sports activity. Second, we describe the main application of CMR in highly trained athletes. Finally, we provide an overview of critical gaps in the literature.

## 2. Normal Ranges for Healthy Cardiac Adaptation—What Do I Mean When I Talk about “Normal Values”?

The first description of cardiac dilatation and hypertrophy due to sports activity in cross-country skiers using percussion goes way back to the dawn of the 19th century [7,8]. The introduction of modern imaging techniques, most importantly echocardiography, has revolutionized our understanding of the athlete’s heart. Indeed, it has remained the primary method to investigate physiological cardiac adaptation due to prolonged and intensive exercise. However, CMR is superior at describing subtle alterations and can ensure the clarification of challenging diagnoses. For this reason, CMR is now an important second-line method of investigation for athletes with the suspicion of structural alterations [9].

The cine CMR sequence has been established as the gold-standard method for quantification of ventricular volumes, function and myocardial mass in the general population [6,10,11,12]. However, to date, the only published meta-analysis focusing on the characteristics of the athletes’ heart using CMR is limited to Caucasian, adult, male athletes competing in endurance and mixed sports [13]. Herein, we summarize the main advances in the field and identify critical gaps in the literature regarding the effect of age, sex, ethnicity, sports exposure and post-processing software disparities.

### 2.1. Age

Cardiac morphology, function and adaptation to exercise changes with age [14,15]. In terms of cardiovascular screening for athletes the recommendation differentiates between young competitive athletes (<35 years) and master athletes (≥35 years) [16]. Adolescent athletes (12–17 years) should be also considered separately, in terms of their adaptation to sports activity due to age-dependent hormonal transition that can potentially unmask genetically determined diseases in this age group [17]. Adolescent athletes have been shown to present only modest increases in left ventricular (LV) cavity size [18], and maximal wall thickness [19] compared to non-athletes. This might suggest that exercise has a different effect on the immature heart, but it may also occur due to lower training intensity and cumulative exercise load at younger ages [18].

Cousergue et al. demonstrated a strong relationship between age-induced changes in LV diastolic function and left atrial (LA) function in healthy young (<35 years) and master (>35 years) athletes using echocardiography [20]. Importantly, Torlasco et al. found key differences in the pattern of adaptation caused by physical activity using CMR. They investigated 237 untrained healthy subjects over a mid-term (6 months) unsupervised physical training, which led to increased ventricular volumes among participants under the age of 35 years, whilst training caused predominantly vascular remodeling among those over 35 years [15]. This is potentially due to the impaired cardiovascular elasticity driven by a reduced number of cardiomyocytes, changes in collagen structure and the reduced responsivity of the heart to sympathetic stimulation as a result of declined cardiac innervation [21,22,23].

### 2.2. Sex

Despite female athletes taking up to half of the whole athletic population [24], the cardiac adaptation in women is still incompletely understood. Sex differences have been described mainly through studies using echocardiography, generally showing that female athletes have a less pronounced cardiac adaptation compared to man [25]. In contrast, D’Ascenzi has recently shown that highly trained women exhibit a relatively larger increase in cavity dimensions compared to men [26]. In a small athletic cohort, Petersen et al. found no sex-specific differences in training effect on LV and RV volumes, mass indices, and ejection fractions, as well as LV to RV ratios of these volume and mass indices [27].

Csecs et al. on the other hand, examined 327 healthy Caucasian athletes using CMR, including 85 (26%) female athletes to investigate the influence of age, sex, body size, sporting type and training volume on cardiac size and function [28]. They found that both adult and adolescent male athletes showed larger mean ventricular volume and ventricular mass compared with their female counterparts. Importantly, in their multivariate model testing for the potential contributing factors of LV mass index, sex and endurance sports were the strongest. Similarly, Maestrini et al. confirmed that Olympic-level male endurance athletes presented higher volumes and LV mass compared to their female counterparts. Interestingly atria dimensions, systolic function and sphericity index did not differ between sexes in this analysis [29]. Of note, these last two studies did not formally test the relative increase in cardiac measures or the interactions between sex and training load.

Indeed, while the overall size of male athlete’s heart is clearly more enlarged than the female’s, the differences in the relative volumetric and mass changes induced by sports activity are still inconclusive [30].

### 2.3. Ethnicity

Data regarding ethnic differences using CMR is scarce, despite the well-documented impact of origin on the cardiovascular response to high intensity exercise [31]. In the worst case scenario, this lack of evidence can potentially prevent timely diagnosis and preventive measures among athletes of less well described ethnic origin [32].

Black athletes present predominantly similar exercise-related adaptation as white athletes, however they generally demonstrate an increased LV wall thickness and a more concentric pattern of hypertrophy compared to white athletes [31,33]. Riding et al. have shown competitive male athletes participating in mixed sports exhibit LV hypertrophy more frequently in cases of African-American/Caribbean (9.5%) and West African (5%) origin compared to athletes with West Asian (0.8%), East African (0%), and North African (0%) ancestry even after accounting for size [34]. This trend is rather similar among female athletes too [35].

Critically, echocardiography data from Malhotra et al. [36] shows that mixed-race soccer players have a greater LV wall thickness than white athletes even after indexing for body surface area. In a study of 3000 mixed race, black and white athletes, they found that black athletes have a greater LV wall thickness compared with both white and mixed-race athletes. Moreover, LV wall thickness over 12 mm was present in 7.1% of black athletes, 5.9% of mixed-race athletes, and 1.3% of white athletes. In the future, classification based on genetic ancestry might enable an even more accurate description of cardiovascular adaptation leading to less racial bias in the general and athletic population alike [37].

### 2.4. Type and Intensity of Sports Exposure

As per the 2020 ESC guidelines, we can differentiate between athletes based on level of sports activity and the type of sports they perform [16]. Comparing elite endurance athletes (sports activity >18 h/week), and regular athletes (sports activity 9–18 h/week) to non-athletes (sports activity <3 h/week) Prakken et al. found that both absolute and body surface area-indexed LV and RV volumes and LV myocardial mass are significantly higher in athletes compared with non-athletes [38]. However, the variation between different levels of exercise is less clearly defined. The relationship between time spent exercising and measures of cardiac remodeling shows an ambiguous relationship, depending to the athletic population in question [28,38].

The differences between sports disciplines are also widely studied, however, it is rather challenging to compare exercise-type related remodeling owing to the differences in the classification of sports in the literature [28,39,40]. In general, endurance sports are associated with ventricular dilatation and eccentric hypertrophy, mixed sports with slightly more modest and balanced dilatation and strength exercise with a more concentric pattern of hypertrophy. However, these are only schematic descriptions and recent data suggest that different sports even within the same discipline might induce certain specific functional alteration or changes in the cardiac deformation [41,42].

In contrast, D’Ascenzi reported that RV volumes and LV mass were independent of sports discipline in their meta-analysis [13]. Of note, the latter study did not differentiate between athletes under and over 35 years and the dataset only contained mixed and endurance athletes.

### 2.5. Post-Processing Sofware and Contouring Methods

Although it is very well known that different contouring approaches have an important impact on cardiovascular measures [43], no study has formally assessed the potential impact of using different post processing software, or contouring methods in healthy athletes.

## 3. Hinge Point Fibrosis—Bad Actor or Innocent Bystander?

The right ventricular insertion points (RVIP) to the anterior and posterior ventricular septum, also known as hinge points, are zones of transition where RV and LV muscular fibers cross each other. In these areas, the presence of isolated focal myocardial disarray has been demonstrated thanks to autopsy studies conducted on hearts with HCM [44,45]. Subsequently, isolated myocardial disarray in the RVIP has been seen as a frequent and non-specific finding both in normal (especially athletes) and abnormal conditions (for instance hypertensive cardiomyopathy or aortic stenosis) [46,47].

The reason why RVIP can present myocardial disarray is still a matter of debate. Given their location and configuration, RVIP seem to be particularly sensitive to ventricular pressure overloading. It has been demonstrated in animal models that a raised RV wall stress for increased pressure loading may lead to incremental biventricular fibrosis, predominantly involving the septal hinge points, probably involving increased growth factors signaling, predominately expressed at the level of RVIP [48,49].

Beside histopathology, the presence of an altered myocardial tissue at ventricular interceptions has also been demonstrated by imaging. LGE confined to isolated RVIP (hinge point LGE or “junctional” LGE) is a common finding on CMR imaging. It has raised interest since its first identification in HCM patients, among whom focal fibrosis in the insertion points has been reported in at least 10% of cases [50,51]. However, neither the presence nor the extent of LGE in these areas have been proven to predict adverse events among HCM subjects. Recent studies suggest that RVIP LGE may present different histological characteristics compared to LGE in other areas, with different prognostic implications. Indeed, autopsy analysis have shown that the RVIP region is composed of expanded extracellular space containing interstitial fibrosis, adipose tissue and disarrayed myocytes, while no signs of scarring as a repair process have been identified. Therefore, it was suggested that RVIP LGE presence in HCM could represent gadolinium storage in an expanded extracellular space with disorganized architecture, rather than replacement scarring [51,52].

Hinge point LGE has also been described in other clinical conditions characterized by RV overload [53]. Interestingly, a recent analysis has reported a high prevalence (up to 40%) of RVIP LGE in patients with dilated cardiomyopathy (DCM), with higher LGE extension in the presence of higher wall stress [54].

While LGE imaging is able to evaluate advanced stages of remodeled myocardial tissue, early stages of RVIP disarray may be more subtle and difficult to detect. Newer imaging techniques such as T1-mapping techniques may be more sensitive for the identification of such changes [55,56].

Beyond these considerations, hinge point LGE is also a common finding in healthy subjects with otherwise normal CMR findings and seems to have no prognostic implications [57]. Furthermore, insertion point LGE has been also observed in around 10% of healthy elderly individuals and may form one of the elements of aging hearts [58].

**Figure 1:**
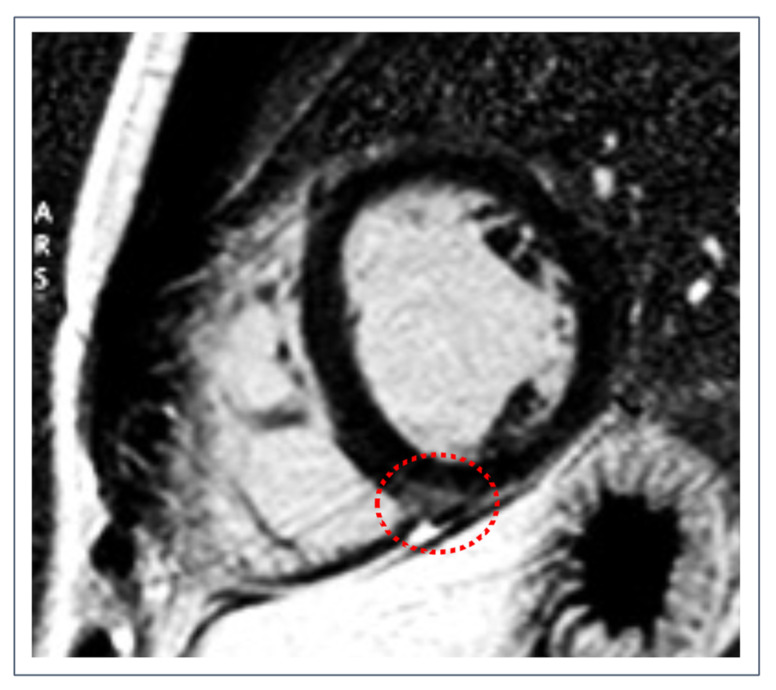
Post-contrast short-axis imaging of a healthy 25-year-old male competitive athlete with isolated RVIP fibrosis (dotted red circle) in the context of an otherwise normal CMR.

Highly trained endurance athletes (Figure 1) showed a ten-fold increase in the prevalence of focal RVIP LGE as compared to control subjects, along with a globally higher myocardial extracellular volume (ECV) values [59]. The prevalence of RVIP LGE among well-trained athletes may reach up to 30% and has been correlated with the cumulative training load and training intensity [60]. As previously seen for a number of pathological scenarios, this correlation between training load and RVIP alterations may be related to the prolonged pressure and volume overload during intensive exercise, which causes tension on the LV/RV insertion points consequently leading to microinjuries visible as spots of LGE. This hypothesis has been partially confirmed by a recent study evaluating CMR parameters in ultra-marathon runners, which found that athletes with RVIP LGE had higher training volume history and higher right ventricular end-diastolic volume index in comparison to those without LGE. These results may suggest a relationship between hinge-point fibrosis and volume overload [61]. Nonetheless, the total amount of LGE did not significantly differ between athletes and sedentary controls, thus making it challenging to understand the role of endurance training in determining LGE. Finally, the long-term prognostic role of isolated RVIP LGE in the subset of otherwise healthy athletes has yet to be investigated.

## 4. Mapping Normal Values and Isolated Mapping Alterations—Pathognomic Marker or Misunderstood Measurement Error?

Emerging CMR techniques including native T1 and T2 mapping, have become more widely available in recent years, offering new opportunities for the non-invasive identification of various cardiac pathologies [62]. Native T1 mapping enables the quantitative assessment of tissue characteristics such as myocardial fibrosis [63], amyloid deposition [64] and lipid accumulation [65]. T2 mapping can detect myocardial edema in various settings including acute myocarditis [66]. Therefore, non-contrast mapping techniques might have an impact on the early detection of diffuse pathologies while maintaining a relatively short scan time and without the addition of contrast material.

In spite of the successful application of the technique for various differential diagnostic questions, the sex, ethnicity and training dependence of native T1 and T2 mapping values remain incompletely understood [60]. It is also unclear how recreational sports activity affects these parameters. Importantly, critical differences within scanner types, mapping sequences, and the lack of universal phantom use can hamper the global standardization of mapping values of in the near future [6,43,62].

Native T1 mapping is generally slightly decreased among young, healthy athletes [67,68,69,70], probably due to the increased myocyte mass (causing expansion of intracellular space) in relation to the extracellular space [68,71]. Meanwhile, other authors reported no difference in T1 compared to less active individuals [72,73]. There is less dedicated research regarding the changes of T2 relaxation time with regard to physical activity. Investigating T2 mapping values of healthy elite athletes and volunteers, Szabo et al. reported no differences between the two groups [70].

Notably, limited studies have already demonstrated the additional value of native mapping sequences to better differentiate pathological alterations compared to standard volumetric measures based on cine images. In a small proof-of-concept study, Gastl et al. demonstrated that T2 mapping and deformation imaging may help distinguish left ventricular hypertrophy caused by physiological remodeling due to sports activity and pathological from due to HCM [74]. Similarly, Swoboda demonstrated key differences between HCM patients and athletes even in subjects with gray zone (12–15 mm) hypertrophy using T1 mapping and extracellular volume measurement [71].

Mapping alterations were widely reported in studies investigating the myocardial involvement after SARS-CoV-2 infection in athletes [75,76,77,78,79]. Importantly, these alterations sometimes occurred without any accompanying sign or symptoms of myocardial damage, raising the question of “subclinical” disease versus measurement error. Other studies performed in the general population highlighted the limits of our understanding of these novel and highly sensitive diagnostic tools [80,81]. Experts warned the CMR community to exercise caution when reporting and evaluating isolated mapping alterations, in order to prevent the overdiagnosis of pathologic findings such as myocardial oedema or fibrosis [81,82].

## 5. Current Evidence of Clinical CMR in the Diagnosis and Management of Athletes

Competitive athletes are a growing population, often (erroneously) considered as “immune” to cardiovascular diseases. Conversely, vigorous physical activity might increase the risk for adverse events up to SCD, especially among subjects with concealed cardiovascular diseases [83].

In the last few years, shared recommendations and pragmatical approaches on athletes’ evaluation have been provided, to distinguish between normality and abnormality [9,84,85]. As mentioned above, CMR use in athletes is rapidly increasing, thanks to the excellent reproducibility, and the large spectrum of morpho-functional information it can provide. In this section, we will look at some of the disease that might be encountered during evaluation of the athlete’s heart.

### 5.1. Hypertrophic Cardiomyopathy

HCM is one the leading cause of SCD among young athletes [86], and its diagnosis in athletes is not always straightforward. The disease may be suspected when LV end-diastolic wall thickness exceeds 15 mm, since few athletes reach this value of hypertrophy as a physiological adaptation to exercise; the diagnostic suspect is reinforced when other characteristics (ECG, echocardiography, family history or symptoms) are found. However, the differential diagnosis becomes more complex in borderline forms of LV hypertrophy (13–15 mm); indeed, a non-negligible proportion of male healthy athletes (especially those of black ethnicity) reach values of end-diastolic wall thickness between 13 and 16 mm, a range defining the so-called “gray-zone” [39,87].

**Figure 2:**
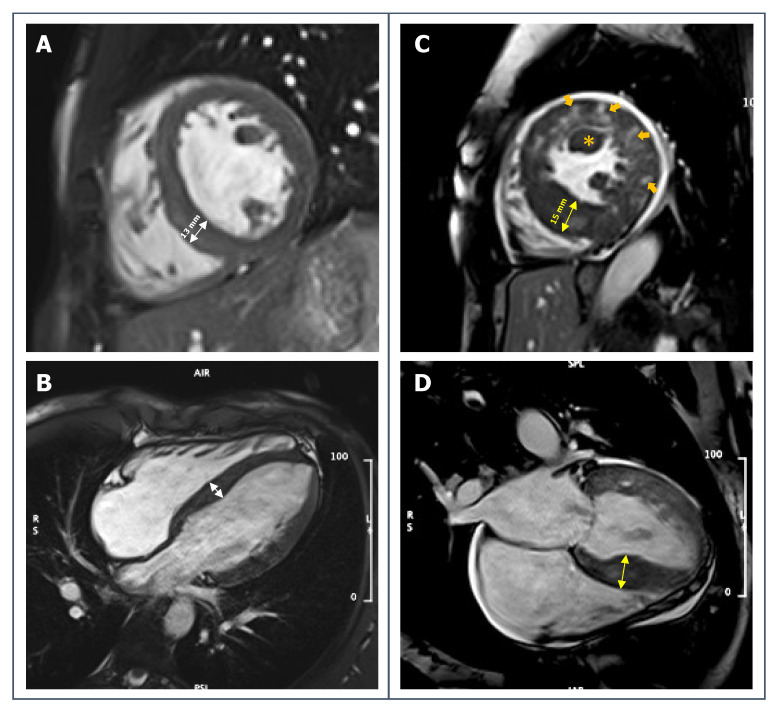
**Left:** short-axis (**A**) and 4-chambers long-axis (**B**) SSFP-images of a 27-year-old male competitive athlete with borderline LV symmetrical hypertrophy (13 mm, white arrows) in the context of mildly increased biventricular volumes and normal biventricular function and no other symptoms or signs of disease. **Right:** short-axis (**C**) and 4-chambers long-axis (**D**) SSFP-images of a 32-year-old male athlete with increased LV wall thickness especially on interventricular septum (15 mm, yellow arrows), relatively small LV cavity obliterating in systole, papillary muscle hypertrophy (orange asterisk); sequences have been acquired after gadolinium injection and inhomogeneous myocardial signal can be appreciated in these images (orange arrows). The athlete had been referred to CMR for ECG abnormalities.

Notably, the current cut-off values for the diagnosis of HCM are based on echocardiographic, and not CMR, studies. However, when measuring the interventricular septum by echocardiography it is common to wrongly include RV trabeculae, thus overestimating septal thickness. Another ambiguous region is the LV apex, where focal hypertrophy can frequently be unrecognized [88,89,90] (Figure 2). For a more individualized definition of hypertrophy, previous studies have proposed a cut-off of LV thickness indexed to end-diastolic volume [91,92]. However, this may not be applicable to athletes performing strength disciplines, as these sports generally lead to a more concentric LV hypertrophy. Moreover, in athletes with apical phenotype HCM, the cavity size may be larger than expected.

ECV is typically decreased in athlete’s heart and increased in HCM [71,93]. Moreover, T2 values and native T1 values seem to be significantly prolonged in patients with HCM as compared to healthy athletes [62,74,93].

**Figure 3:**
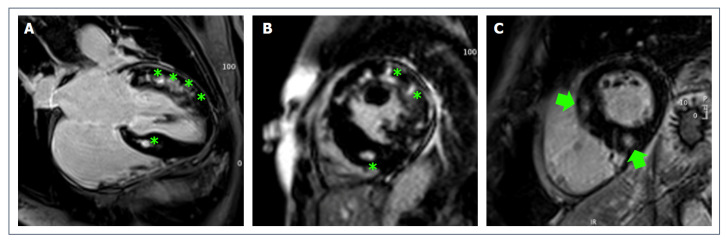
(**A**,**B**): post-contrast imaging (apical 4-chambers view and mid short-axis view) of a 32-year-old male athlete with a diagnosis of HCM with diffuse areas of patchy non-ischemic LGE (green asterisks). (**C**): short-axis view post-contrast imaging of a 41-year-old female ex-athlete with HCM showing areas of LGE in the basal anteroseptal segment and inferior RVIP (green arrows).

Contrast-enhanced CMR has been applied to HCM both as an adjunctive diagnostic technique and as a tool for SCD risk stratification [51,94,95]. LGE is present in more than 50% of patients with HCM [96], while it should not be observable in healthy athletes [59,60]. As previously described, the presence of isolated RVIP LGE in HCM does not seem to have prognostic meaning; conversely, the presence of LGE outside hinge-points is associated with increased risk of SCD and overall mortality [51,94,95,97] (Figure 3). Previous studies have also proposed specific cut-off of LGE amount to help in the risk stratification [90]. However, specific studies on the prognostic value of these risk features in competitive athletes are still missing.

CMR is used for risk stratification according to the 2020 ESC Guidelines on sports cardiology. Importantly, the presence of extensive (≥15% of LV myocardium) LGE may identify individuals at increased risk of ventricular tachyarrhythmias and SCD [95,98,99,100]. In borderline cases, detraining for approximately 3 months may be used to differentiate between the athletes heart and HCM [101]. However, in the absence of high risk features a detraining would not have a consequence on decision-making but might potentially have unnecessarily negative effects on the athlete’s life.

### 5.2. Dilated Cardiomyopathy

Enlargement of LV cavity is a part of the expression of cardiac adaptation to intense exercise, particularly that with high isotonic/dynamic components. The entity of LV dilatation should therefore be interpreted considering the sports discipline, the body size, the contextual LV hypertrophy, and the harmonic dilatation of the other cardiac chambers [102]. Up to 40% of elite athletes have an increased LV end-diastolic diameter and, in a minority of them, the dilatation reaches values >60 mm with a mildly impaired ejection fraction [39,103]. In these cases, the distinction with mild forms of cardiomyopathy is challenging and of outmost importance, as DCM accounts for up to 8% of SCD in athletes [104,105].

**Figure 4:**
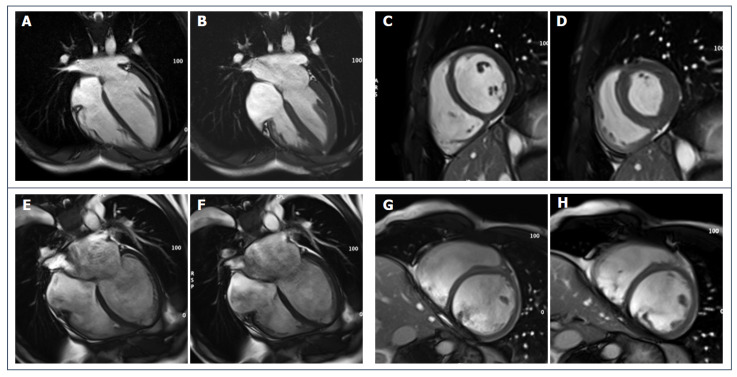
(**A**–**D**): 28-year-old male endurance athlete; SSFP cine sequences (**A**,**C**): diastolic frames show mild LV dilatation with harmonic dilatation of the other cardiac chambers and preserved systolic function (**B**,**D**): systolic frames. (**E**,**F**): SSFP sequences of a 34-year-old male patient with DCM; LV is severely dilated, spherical and moderately dysfunctional (**E**,**G**: diastolic frames, **F**,**H**: systolic frames).

Echocardiographic parameters such as diastolic indexes, strain analysis, LV wall thickness and ejection fraction (at rest and during stress), along with family history, ECG, evaluation of arrhythmias, are the basic diagnostic tools [9]. CMR can offer additional value by providing accurate and reproducible measurements (Figure 4).

Recently, the myocardial deformational mechanics by feature-tracking CMR (FT-CMR) have been proposed as an aid in the distinction between adaptation to exercise and pathological dilatation, since DCM has different deformational characteristics as compared to both sedentary and athletes hearts [73]. Moreover mapping technique can be useful in this setting: native T1, ECV and T2 values are all significantly increased in DCM (even in early stages) as compared to normal hearts, and athletes hearts [72].

**Figure 5:**
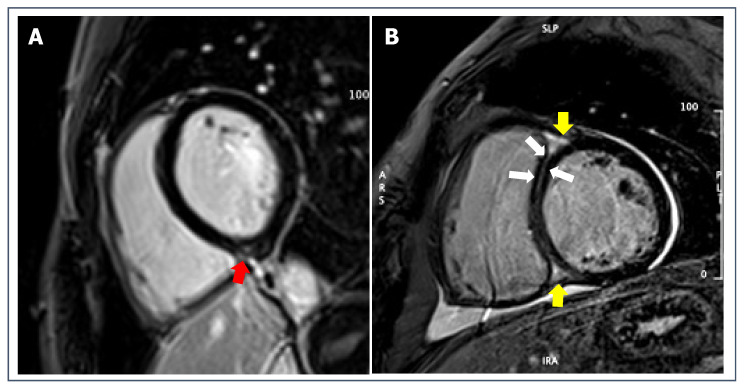
LGE short-axis sequences of a 28-year-old male endurance athlete with athlete’s hearts (**A**) and of a 34-year-old male patient with DCM (**B**). In the first case, mild LGE signal is seen in the inferior RVIP (red arrow). In (**B**), a thin LGE stria can be appreciated in the interventricular (IV) septum (white arrows), and a more pronounced LGE storage is visible in the anterior and inferior RVIP (yellow arrows).

The main additional value of CMR in this field is the evaluation of fibrosis. The presence of nonischemic LGE (more typically, but not exclusively, as a mid-wall septal stria) is more consistent with DCM, even if the absence of LGE does not exclude the disease [106] (Figure 5). LGE may also be found in the early stages of DCM and is associated with a worsened clinical outcome and the risk of arrhythmic events regardless of the severity of LV dysfunction [107]. Barison et al. have shown that the amount of LGE correlates with major arrhythmic endpoints. They proposed a cutoff of LGE extent at >13% [108]; however, it needs be proven if the same data can be applied to athletes.

### 5.3. Arrhythmogenic Cardiomyopathy

Arrhythmogenic cardiomyopathy (ACM) is one of the leading causes of SCD in athletes; moreover, it is well known that intense and repetitive exercise training can accelerate and worsen the course of the disease [109]. The diagnosis of ACM is complex and requires a multiparametric approach [110]; electrical changes, both in the general population and in athletes, the morphological changes may proceed over a long time period [111]. Echocardiography has important limitations in identifying the disease, especially in its early stages. This is true both for the right/biventricular forms (as the complex geometry of RV can be hard to visualize) and for left variant forms, since the disease typically spares the endocardium that contributes most to systolic function and kinetics. Therefore, modern approaches to the diagnosis strongly rely on CMR.

**Figure 6:**
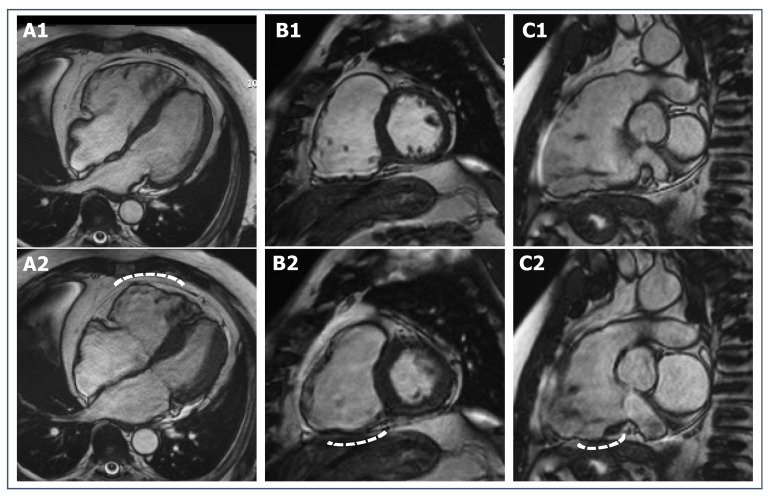
SSFP cine CMR sequences of a 33-year-old female ex-athlete with arrhythmogenic right ventricular cardiomyopathy. RV dilatation and dysfunction can be appreciated through diastolic and systolic frames ((**A1**,**A2**) for 4-chamber view, (**B1**,**B2**) for short-axis view, (**C1**,**C2**) for RV vertical long-axis view, respectively), as well as wall motion abnormalities (white dotted lines).

The new diagnostic criteria for ACM [110] requires the presence of at least one morpho-functional (dilatation, kinetics abnormalities and/or dysfunction) or structural (fibrous or fibro-fatty tissue on CMR or endomyocardial biopsy) criteria. In athletes, the cut-off values for RV dilatation proposed in the 2010 criteria [112] may lack specificity because significative RV dilatation can be observed as a physiological adaptation to endurance exercise and increased prolonged RV wall stress [113]. Proper reference values for RV volume in athletes have been reported to differentiate physiologic from pathologic RV dilatation in athletes [13]. Moreover, physiological RV remodeling should be harmonic, different to the predominant dilatation of the outflow tract in ACM patients [114]. RV ejection fraction is frequently reduced in ACM and generally preserved in athletes, although CMR studies have shown that mildly reduced RV function can be observed in up to 5% of elite athletes [115] (Figure 6).

**Figure 7:**
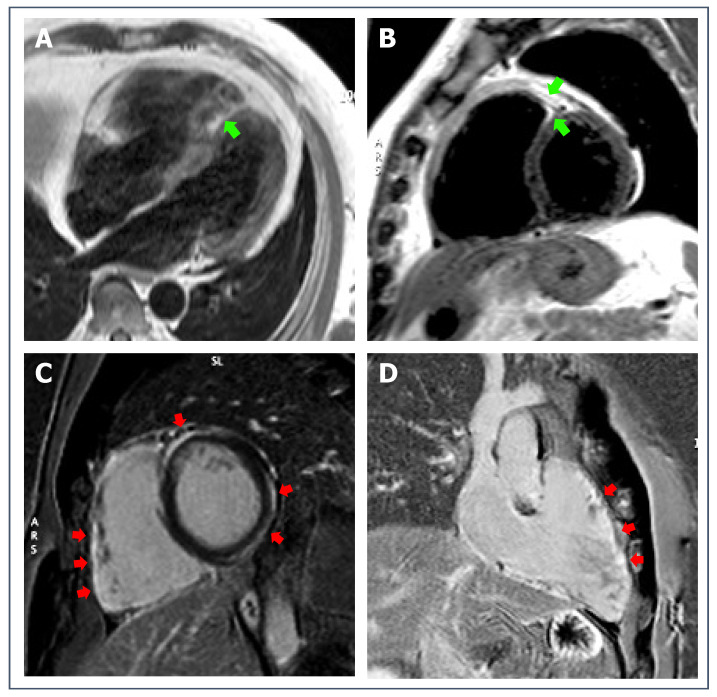
43-year-old patient with biventricular ACM. T1-weighted sequences (**A**), 4-chambers view, and (**B**), short-axis view) show mild signs of fatty infiltration (green arrows). Post-contrast sequences (**C**), short-axis view, and (**D**), right heart 2-chambers view) reveal non-ischemic LGE involving both RV and LV walls (red arrows).

Structural evaluation is crucial for the diagnosis of ACM regardless of the exercise level since the disease is characterized by a fibrous (or fibro-fatty) replacement of myocardium. LGE is observed in the majority of cases of ACM. The typical pattern of LGE in left-variant ACM is a subepicardial stria involving more commonly the lateral or infero-lateral wall; concomitant fatty infiltration is often observed in the same regions, even if it is neither specific nor necessary for the diagnosis [116,117] (Figure 7). The assessment of LGE or fatty tissue in RV is challenging because of its thin wall; LGE imaging can be combined with regional wall motion assessment to enhance the sensitivity of CMR [118].

Recently, RV strain analysis with FT-CMR has been applied to the field of athletes with suspected ACM, demonstrating good discrimination between athlete’s heart and right-ventricular ACM [119].

### 5.4. Non-Ischemic Scar

An isolated non-ischemic left ventricular scar (NLVS) is defined as the presence of LGE with a non-ischemic pattern in the LV myocardium in the absence of other distinctive signs of cardiomyopathy. It is an emerging substrate of ventricular arrhythmias and SCD occurring during effort [120,121,122]. NLVS has been originally interpreted as the result of a previous concealed myocarditis, even if it may be the expression of a biventricular or left variant ACM; in the absence of family history, genetic data or other specific features of ACM, the distinction between the two etiologies is challenging. Even the presence of concomitant fatty infiltration is not exclusive of ACM, as it can be part of the natural process of repair after myocardial injury.

**Figure 8:**
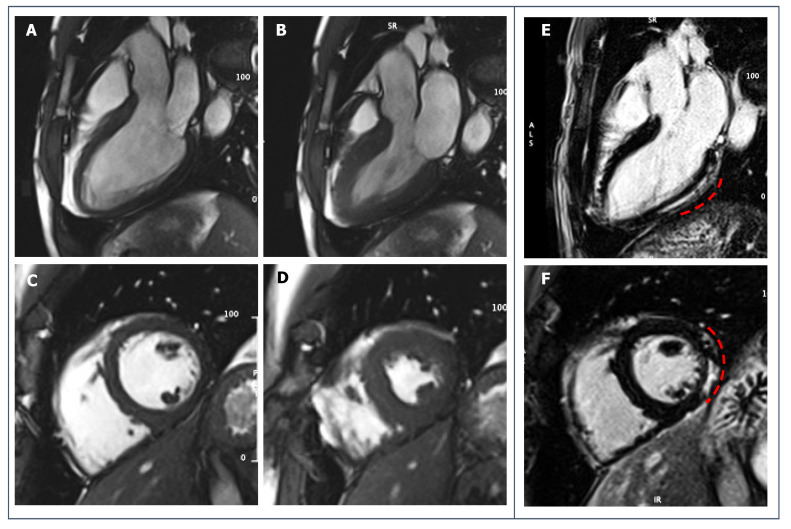
39-year-old male football player with uncommon ventricular arrhythmias. No systolic dysfunction or wall motion abnormalities are seen on SSFP cine-sequences (**A**,**B**): diastolic and systolic frames of 3-chambers view; (**C**,**D**): diastolic and systolic frames of short-axis view. (**E**,**F**): post-contrast imaging shows a sub-epicardial stria of LGE involving the basal and middle portion of infero-lateral LV wall (red dotted lines).

CMR offers the possibility of identifying myocardial fibrosis even where other techniques fail. Indeed, since non-ischemic scar by definition spares the endocardium, no wall motion abnormalities or systolic dysfunction are seen, except in cases of extensive scar replacement (Figure 8). ECG abnormalities such as T-wave inversion or low QRS voltages in the limb leads can raise the suspicion of a concealed myocardial fibrosis, but they are not sensitive enough to exclude the disease [123]. When the suspect is raised, CMR is essential for the diagnosis even when second level testing (ECG, echocardiography) is normal.

In caseswe of an inflammatory etiology of the NLVS, CMR also offers the possibility to display the presence of myocardial edema thanks to T2-weighted sequences and T2 mapping technique indicating an ongoing inflammation [124,125].

The presence of non-ischemic LGE is a marker of possible arrhythmic risk per se. Recently, it has been suggested that LGE amount, but also pattern and localization (for examples, ring-like scar) may carry higher arrhythmic risk [126]. Moreover, the concomitant presence of fatty metaplasia has been associated with negative outcomes [127]. However, the clinical meaning of persistent LGE in an athlete after an acute inflammatory process, in the absence of LV dysfunction or arrhythmias, is still a matter of debate, and it has to be established if there is a specific amount of LGE above which restriction to sports activity should be recommended [128,129].

### 5.5. Left Ventricular Non-Compaction

Left ventricular non-compaction (LVNC) is characterized by prominent myocardial trabeculations and a thin compacted myocardial layer. The proposed diagnostic criteria rely on the evidence of a high non-compacted to compacted ratio on echocardiography or on CMR [130,131,132,133]. However, fulfilling these morphologic criteria per se has not been associated with adverse LV remodeling or outcome [134]. It is debated whether LVNC should be categorized as an independent disease or not. Critically, there is a genetic and morphological overlap with well described diseases such as DCM. Therefore, categorizing potential dilated LVNC cases as DCM with hypertrabeculation is probably more helpful in clinical practice.

On the other hand, high preload conditions including physiological adaptation to pregnancy or intensive exercise are also associated with increased LV trabeculation. Indeed, almost 20% of asymptomatic athletes can have increased LV trabeculation and up to 8%, in particular those of African or Afro-Caribbean origin, fulfil the criteria for LVNC [135,136,137]. Whether hyper-trabeculation in athletes is a form of incomplete expression of LVNC or just a form of cardiac adaptation to exercise is still uncertain. Although the latter seems more likely, studies on athletes with increased trabeculations have shown that generally this pattern was not associated with LV dysfunction or family history of cardiomyopathy [61]. Key features that should suggest cardiac pathology in the context of LVNC are ongoing cardiac symptoms, decreased systolic function, and a family history of heart failure or SCD [16].

**Figure 9:**
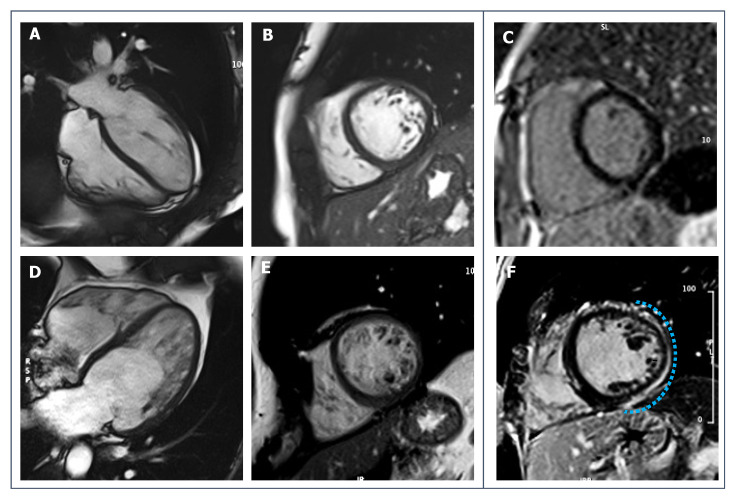
(**A**–**C**)**:** 19-year-old healthy male runner with hypertrabeculation of LV in the context of an otherwise normal CMR. (**D**–**F**): 23-year-old male athlete with a diagnosis of LVNC (later confirmed with genetic testing) with evidence of non-ischemic LGE on post-contrast imaging (blue dotted line).

A complete and multimodal approach is essential for the diagnosis of LVNC, especially in athletes. In suspected cases of LVNC, CMR should be performed to confirm hyper-trabeculation, to better assess LV kinetics and function and to detect areas of LGE that are suggestive for cardiomyopathy [138] (Figure 9). Importantly, the application of published LVNC criteria among athletes is both incorrect and potentially detrimental to patient care.

LGE presence may be seen in LVNC, but the sensitivity is not particularly high [139]. However, its presence may predict adverse outcomes in terms of heart failure and arrhythmias. The few other known predictors of events are LV dilatation and reduced ejection fraction, and a thinned compacted myocardial layer [140,141,142,143].

Recently, mapping studies on LVNC have shown that higher ECV values correlate with worsened outcomes [144]. FT-CMR reports have demonstrated that strain is impaired in patients with LVNC in comparison to patients with normal LV phenotype [145] and that this reduction correlates with the degree of ejection fraction reduction [146]. So far, none of these techniques have been specifically studied in the context of athletes.

### 5.6. Anomalies of the Coronary Artery Origin

**Figure 10:**
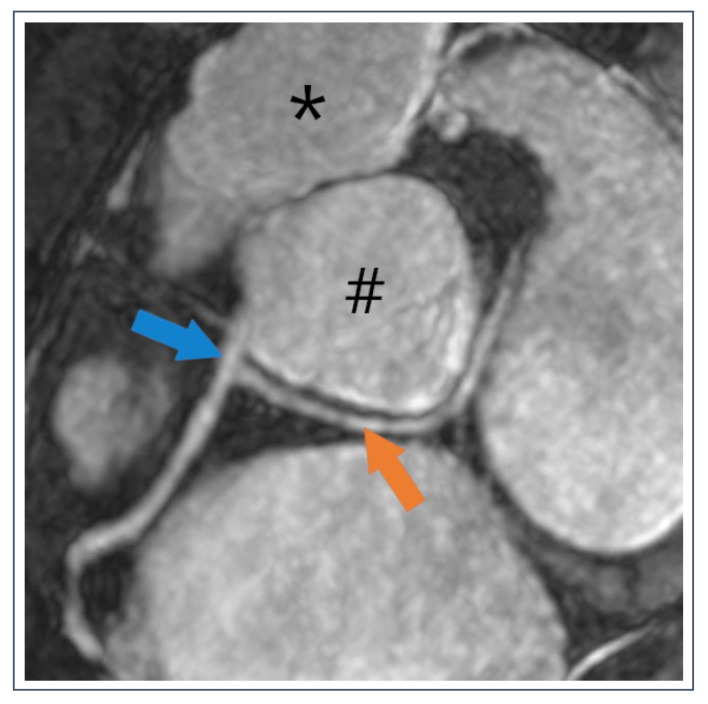
54-year-old recreational athlete with anomalous circumflex artery (CX) running behind the aorta (#). The CX (orange arrow) originates from the right coronary artery (blue arrow) and has a retroaortic course.

**Figure 11:**
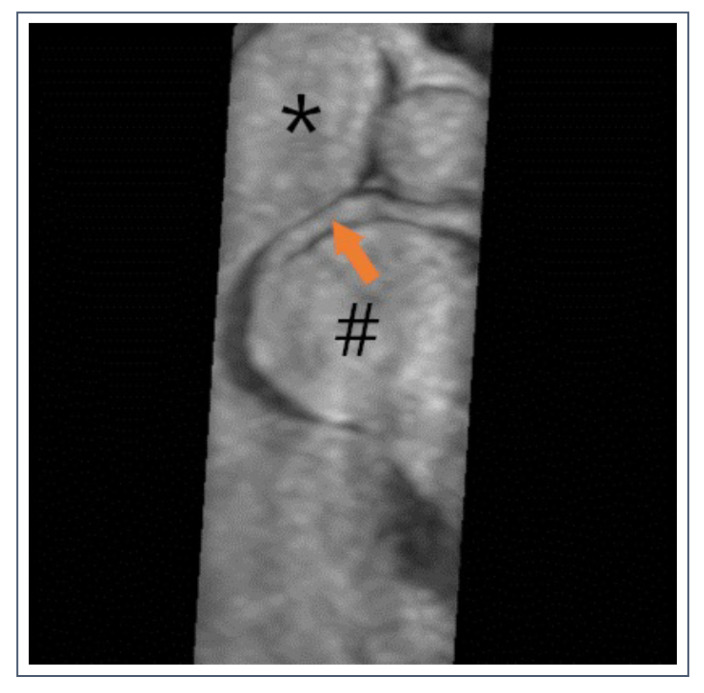
36-year-old pentathlete with anomalous right coronary artery (RCA). Dominant RCA (orange arrow) originates from the left sinus Valsalva and runs between the aorta (#) and the truncus pulmonalis (*).

The estimated prevalence of anomalies of the coronary artery origin (ACAO) in the general population is low; nevertheless, it is a well-recognized substrate of SCD in athletes [147]. Higher risk of SCD is carried by ACAO with hemodynamic impact, such as the origin from a wrong aortic sinus with inter-arterial course, or, more uncommonly, left/right coronary artery origin from the pulmonary artery (ALCAPA/ARCAPA) [148,149]. In athletes with a suspicion of ACAO, advanced morphological imaging is recommended [9], by using coronary computed tomography (CCT) or CMR angiography that can depict coronary origins and their proximal course [150,151]. CMR angiography may be a valid alternative to computed tomography in younger athletes with a low risk of coronary artery disease, due to radiation concerns and the possibility of being performed without the use of contrast agents (Figure 10 and Figure 11). CMR angiography can detect the ectopic origin and the course of a coronary artery and quantify the degree of arterial compression thanks to cross-sectional views [95]. Moreover, CMR offers additional information on biventricular function or myocardial viability. However, so far computed tomography remains the first choice in elite athletes or in those with an increased risk of coronary artery disease, due to the possibility of visualizing the atherosclerotic burden [152]. Beyond morphological evaluation, functional assessment to detect exercise-induced ischemia is needed for clinical management and advice on sports participation. Stress echocardiography, nuclear imaging, coronary artery angiography are classically used, depending on the age and risk profile of the athlete. Stress-perfusion CMR has been shown to have good sensitivity and specificity for the non-invasive assessment of myocardial ischemia in the adult [153,154,155] and pediatric [156,157,158] population, with a higher spatial resolution compared to other techniques and a good correlation with invasive functional tools such as fractional flow reserve.

**Figure 12:**
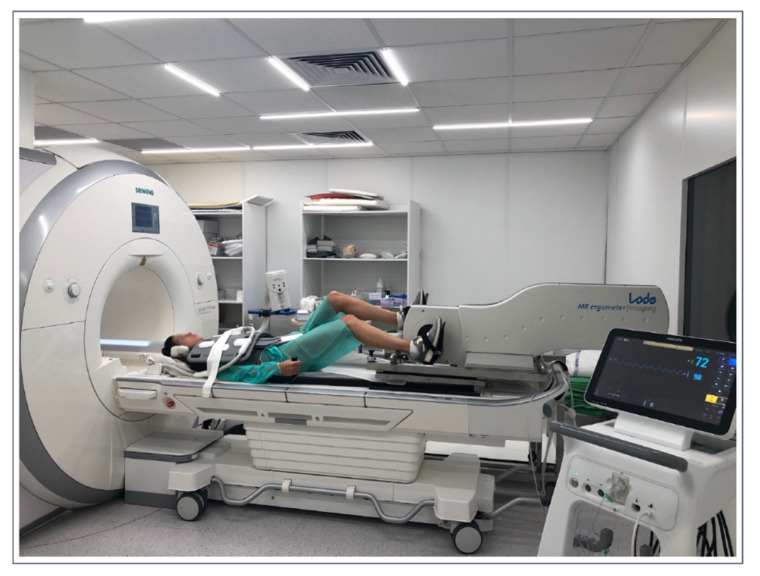
Healthy volunteer is preparing for the stress CMR test.

The use of the novel CMR conditional ergometer allows an even more physiological approach to detect hemodynamic relevance of anomalous coronary arteries by analyzing regional wall motion during physical exercise. This approach is promising and could be superior to stress echocardiography (especially in patients with poor acoustic windows), however its routine clinical use is not yet fully established. Cardiac motion artefacts at high heart rate as well as breathing artefacts and chest motion artefacts limits image quality, but the improvement of image acquisition techniques (e.g., compressed sensing) will likely extend the possibilities in this field (Figure 12) [159].

Finally, evaluation of LGE can offer a demonstration of a previous myocardial ischemia and an aid for risk stratification.

## 6. CMR Technical Difficulties and Pitfalls

As demonstrated in the previous sections a wide variety of CMR sequences offer a detailed description of the heart making it an imaging modality of increasing popularity. However, CMR is also prone to certain technical difficulties and pitfalls that may entail important limitations. The detailed description of CMR artefacts is beyond the scope of this review, and here we refer to source documents addressing these issues [160]. It should be noted that most clinically used CMR sequences require breath-hold and low premature ventricular beat burden during data collection as they are prone to motion artefacts. Although, novel sequences using artificial intelligence are already employed to produce high-quality images for a wider range of patients. These new tools have the power to further improve and democratize the use of CMR.

Untested or low interobserver variability can cause issues during post-processing as well as different contouring methods. It has been long debated if we should include papillary muscles into the compact myocardium or use a simplified contouring technique excluding the papillary muscles and trabeculae [43]. Apart from significantly reducing segmentation time, emerging machine learning tools might also help standardize these contouring protocols in the future [160]. Caution should be taken during LGE characterization, too [43]. As an example, a septal LAD branch or the aortic outflow tract might be misinterpreted as pathological LGE. Ideally, during the evaluation of LGE, two separate, perpendicular views should confirm the presence of LGE.

## 7. Gaps in Knowledge and Future Directions

CMR plays a key role in the identification of cardiovascular disease in athletes, and its application is growing fast. In order to create equal opportunities for diagnosis among athletes, we must tackle several issues. Most evidence on athletes come from the male population. Therefore, studies are needed on female athletes’ heart, as it is known that the pattern of remodeling follows relevant sex differences. Moreover, the validation of normal ranges for physiological heart adaptation among all racial groups using CMR is key to promoting unbiased diagnosis making.

Research should focus on establishing robust normal ranges and cut-off values with regards to less well represented groups in the literature. Clear descriptions of standard operating protocols on how metrics were derived should be prioritized and well documented. To enable the clarification of pathological alteration in lower throughput centers, these normal ranges should be openly available to all.

The increasing use of CMR in the pre-participation screening of competitive athletes is partially limited by the higher cost and lower availability in comparison to echocardiography, as well as the impact of possible false-positive results such as the evidence of a focal area of LGE of uncertain meaning.

Myocardial scarring has been included in the novel European Society of Cardiology guidelines [16] as a criteria for exercise recommendation in different pathological conditions for the first time. The adverse effect of the extent of LGE is probably best established among DCM and HCM patients, although there is still a lack of information regarding athletes. LGE extent over 20% of the myocardium is defined as extensive, warranting a ban from high-intensity sports activity, however there is little data in terms of mid-to-long term outcomes of athletes with any type or amount of scarring.

## Data Availability

Not applicable.
